# Semantic Indexing of Medical Learning Objects: Medical Students' Usage of a Semantic Network

**DOI:** 10.2196/mededu.4479

**Published:** 2015-11-11

**Authors:** Nadine Tix, Paul Gießler, Ursula Ohnesorge-Radtke, Cord Spreckelsen

**Affiliations:** ^1^ Division of Knowledge-Based Systems Department of Medical Informatics RWTH Aachen University Aachen Germany; ^2^ Center for Audiovisual Media Medical School RWTH Aachen University Aachen Germany

**Keywords:** semantic net, usability evaluation, semantic indexing, learning objects, medical education

## Abstract

**Background:**

The Semantically Annotated Media (SAM) project aims to provide a flexible platform for searching, browsing, and indexing medical learning objects (MLOs) based on a semantic network derived from established classification systems. Primarily, SAM supports the Aachen emedia skills lab, but SAM is ready for indexing distributed content and the Simple Knowledge Organizing System standard provides a means for easily upgrading or even exchanging SAM’s semantic network. There is a lack of research addressing the usability of MLO indexes or search portals like SAM and the user behavior with such platforms.

**Objective:**

The purpose of this study was to assess the usability of SAM by investigating characteristic user behavior of medical students accessing MLOs via SAM.

**Methods:**

In this study, we chose a mixed-methods approach. Lean usability testing was combined with usability inspection by having the participants complete four typical usage scenarios before filling out a questionnaire. The questionnaire was based on the IsoMetrics usability inventory. Direct user interaction with SAM (mouse clicks and pages accessed) was logged.

**Results:**

The study analyzed the typical usage patterns and habits of students using a semantic network for accessing MLOs. Four scenarios capturing characteristics of typical tasks to be solved by using SAM yielded high ratings of usability items and showed good results concerning the consistency of indexing by different users. Long-tail phenomena emerge as they are typical for a collaborative Web 2.0 platform. Suitable but nonetheless rarely used keywords were assigned to MLOs by some users.

**Conclusions:**

It is possible to develop a Web-based tool with high usability and acceptance for indexing and retrieval of MLOs. SAM can be applied to indexing multicentered repositories of MLOs collaboratively.

## Introduction

Medical learning objects (MLOs), classical sources (eg, textbooks), and digital media support medical teaching and learning [1]. Maloney et al [2] showed that health professional learners regard Web-based repositories as the preferred learning source. To improve the integration and reuse of existing MLOs in the course of medical education, they need to be systematically collected and organized [1,3]. Semantic indexing of MLOs is a prerequisite of useful repositories of MLOs, and semantic networks (SNs) provide a sound basis for consistent semantic indexing [4]. While SNs have worked their way into medical classification systems and e-learning, the usability of those networks has been sparsely investigated [5,6].

### Project Context

#### Multimedia Learning Objects of the Aachen Emedia Skills Lab

Over the last few years, the Center of Audiovisual Media of the Aachen Medical School has implemented and maintained the emedia skills lab as an interdisciplinary repository of MLOs. The MLOs of the emedia skills lab spread from traditional media (text files, pictures, etc) to modern digital media (videos, podcasts, complete online classes, etc). The MLOs support blended learning and self-studies in addition to the regular classes and thus increase learning flexibility [3]. Additionally, some of the MLOs are integrated in compulsory modules of the local curriculum and are therefore used regularly by learners and medical teachers.

#### Semantically Annotated Media Project

SAM is a Web app based on an SN (see section on Semantic Indexing), which supports MLO retrieval, connects learning resources of similar content, and presents them together [7]. At present, SAM is used for indexing the MLOs of the emedia skills lab. Nonetheless, SAM provides Web services, which enable linking of MLOs organized in different repositories. SAM implements the structure of a Simple Knowledge Organization System (SKOS) [8]. The system was designed as platform that is “opportunistic” towards the terminological knowledge base (the SN used by the system); that is, SAM’s SN can be further developed or completely exchanged. SAM is currently under consideration by other German medical schools.

**Figure 1:**
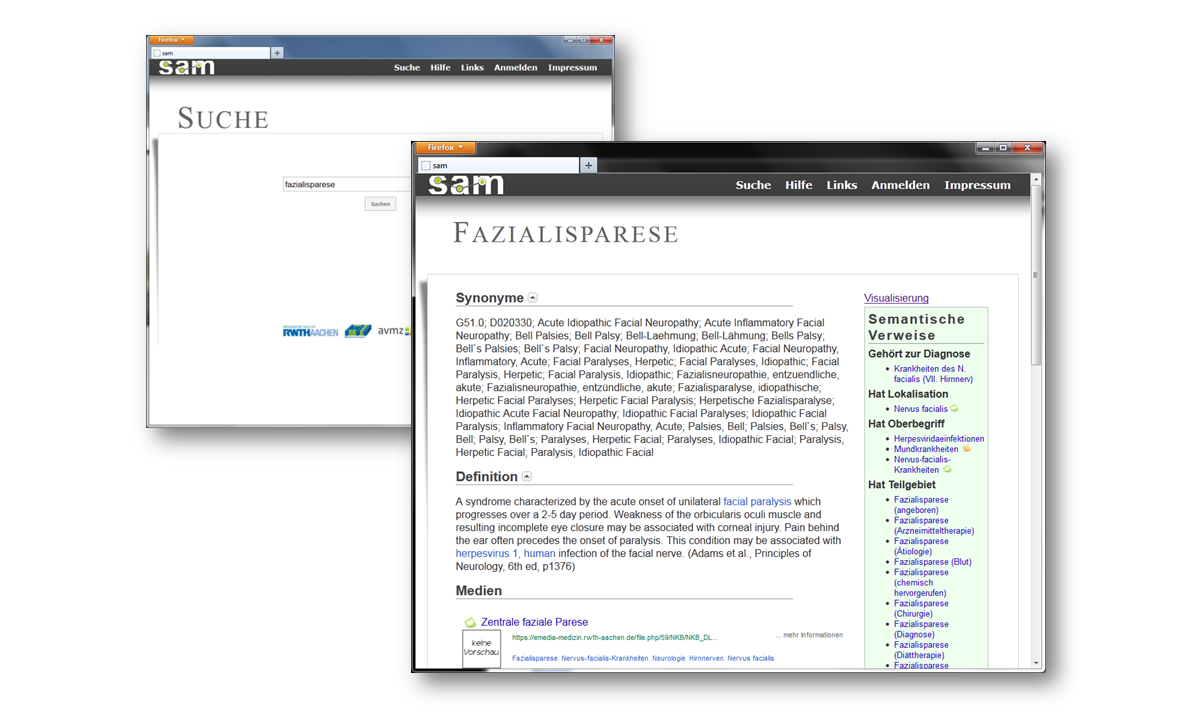
Screenshots of SAM: Search interface and result page contain the SN links (green box).

As an entry point, SAM provides a simple search form equipped with autocomplete functionality. Additionally, an extended search interface allows an easy use of Boolean combinations of search terms and the definition of simple filters. Based on an initial request by the user, SAM generates result pages that show MLOs fitting the query, but more importantly present a part of the SN relevant to the query. Thus, users may explore MLOs directly or switch to the network and explore the space of related concepts, the (synonym) terms associated with a given concept, the semantic links between the concepts, and different MLOs indexed by the SAM concepts, respectively. If the user selects an MLO, SAM presents the link to the MLO in the repository (emedia skills lab) but also gives a short description of the MLO, the set of keywords indexing it, and a list of media with a similar keyword profile. Users may collect the MLOs of their interest following a “cart” metaphor known from online shops. The user’s set of selected MLO items can then be used for retrieving similar MLOs, where the similarity is dynamically calculated from the combination of selected items. Due to the app context, the main language of SAM is German, but SAM also includes English definitions for the MeSH concepts included. See Figure 1 for screenshots of SAM.

#### SAM Semantic Network

SAM is based on existing and established classifications, namely the Medical Subject Headings (MeSH) and the International Classification of Diseases (ICD). The concepts of both classification systems were merged while preserving the taxonomic structure. In addition to generic associations, cross references between the concepts were established based on semantic relations of the Unified Medical Language System (UMLS). At present, SAM covers more than 2,810,000 links between more than 180,000 main concepts. The concepts include subconcepts derived from MeSH terms combined with MeSH qualifiers (eg, “Heart – abnormalities” as a subconcept of “Heart”).

### Background

#### Semantic Indexing of Medical Learning Objects

Assigning textual descriptors to MLOs characterizing their content is challenging with respect to using characteristic descriptors uniformly and consistently. Using a controlled vocabulary (ie, a standardized collection of keywords) supports consistent indexing. Tools like MetaMap [9] or the Medical Text Indexer [10] were implemented to identify suitable keywords to content starting from a given text by adopting language processing and terminological knowledge. Pure keyword indexing lacks associations between objects assigned to different but semantically related keywords (a learner searching media assigned to “appendicitis” clearly may be interested in a video linked to “appendectomy”). As a step forward, SNs (ie, collections of concepts interlinked by meaningful relations) are means for indexing and linking information objects and can support orientation in large information spaces. Cimino et al already saw the potential of SN for reducing cognitive overload of medical hypertexts as early as 1992 [11]. Semantic indexing of medical content is used in different fields of application, for example, in health-related Web resources [12,13]. SNs in e-learning enable a fast and precise search of MLOs as well as organizing and personalizing an e-learning environment [5].

#### Usability

The International Organization for Standardization (ISO) standard ISO 9241-11 defines usability as “The extent to which a product can be used by specified users to achieve specified goals with effectiveness, efficiency, and satisfaction in a specified context of use” [14]. The IsoMetrics usability inventory, a usability inspection method, is a questionnaire based on ISO 9241-10, Part 10 [15]. IsoMetrics was designed according to seven dialogue principles [16]: (1) suitability for the task, (2) self-descriptiveness, (3) controllability, (4) conformity with user expectations, (5) error tolerance, (6) suitability for individualization, and (7) suitability for learning. There is a set of questions for each of those principles with five answers for each question and an option for “no answer”.

Another method to evaluate usability is usability testing. The main aspect of usability testing is the involvement of the target users and the observation of their interactions [17,18]. The users walk through a typical usage scenario of the software, and they are observed by experts on different aspects such as the time they take to complete the task and the number and types of errors they make [19]. Also the users can be monitored, for example by thinking aloud combined with video observance [20]. Usability testing is more costly and time-consuming than usability inspection methods.

Nielsen considered both methods, usability inspection methods and usability testing, as highly appropriate for software usability evaluation [21]. They are the most frequently used procedures according to Hollingsed and Novik [22].

Sandars showed that while usability testing is well established in other disciplines, it has not found its way into medical e-learning [23]. Sandars and Lafferty also pointed out that the usability testing of an e-learning tool should be carried out under the conditions that it is typically used in [24].

#### Long Tail

Long tail is a phenomenon that appeared through Web techniques. Anderson coined the term “long tail” in economics [25], where selling large numbers of very specific products—each in relatively small quantities—could lead to significant profit. O’Reilly stated that a long-tail effect is typical for collaborative Web apps (Web 2.0) [26], which support both the few commonly shared interests (blockbusters) and additionally the huge amount of very specific ones (the long tail). The effect is illustrated, for example, by the power-law shaped popularity rates in social networks [27]. Being a genuine Web-based approach, crowd-based semantic indexing of digital content illustrates characteristics of a long-tail phenomenon. When many users propose index terms for given content, a long tail of keywords used by only a few persons may arise by erroneous choice, but could also be due to aspects relevant for comprehensive indexing, but noticed by only a few users. As a characteristic aspect of user behavior, we explored this issue for the SAM app.

### Statement of Purpose

This study aimed at assessing the usability of SAM when used as a tool for accessing and indexing MLOs. Clearly, the usability of SAM has two aspects: (1) The usability of SAM’s user interface (which can be adopted by any MLO repository providing deep HTML-links, and which can operate on any SKOS-conform SN), and (2) the suitability of the currently used SN for accessing and indexing MLOs.

Based on typical tasks (scenarios), the study should uncover typical usage patterns of students using SAM for browsing, searching, and indexing MLOs. From those results, we intend to derive recommendations for apps improving access to MLOs by semantic indices.

### Rationale for the Approach to the Problem

We decided to use a scenario-based approach in order to evaluate SAM. The participants were students of the Evidence-Based Medicine (EBM) class in their second year of medical school-target users for SAM.

## Methods

### Study Design

For this study, we chose a mixed-methods approach. Lean usability testing was combined with usability inspection by having the participants complete four typical usage scenarios before filling out a questionnaire. The questionnaire was based on the IsoMetrics usability inventory because of its widespread use and also because it is advised for a large group of participants to ensure accurate analysis [15]. Here, only the questions from the areas of “suitability for the task” and “suitability for learning” were used. Some extra questions regarding the use of SAM were added, which yielded a total of 19 usability questions. At the end of the survey, there were three open questions for positive and negative feedback as well as improvement suggestions for SAM as a basis for qualitative analysis.

The EBM course is a mandatory module of the curriculum (attended by the first and the second half of each student cohort in the 4th and 5th semester, respectively). Information retrieval including a scenario-based introduction to SAM is a permanent part of the EBM course introduced 2 years before this study was conducted and has continued ever since. Participation in the study was voluntary (see section on Ethical Issues).

**Figure 2:**
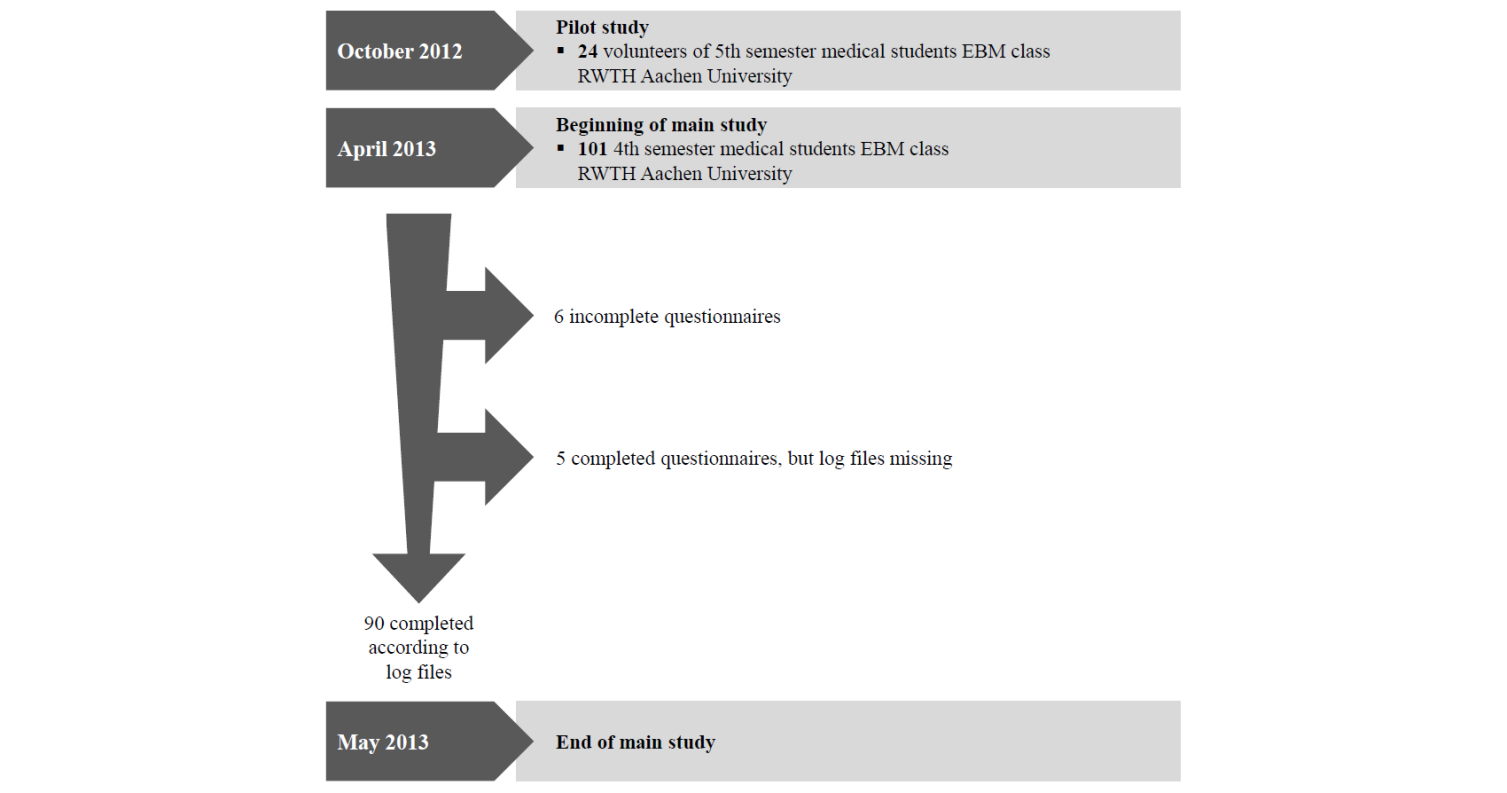
Course of study.

Figure 2 shows the course of the study. A pilot study (n=24) took place in October 2012. As a consequence of the pilot study, one more usability question regarding SAM was added and the four scenarios were established. The medical students assigned to take the EBM class in their 4th semester in May 2013 (N=101) were informed about the study and asked to participate. Of these, 95 participants consented and completed the questionnaires. According to the log files, only 90 students accomplished all four search queries given by the scenarios. All participants used computers provided by the university, and logging was based on the computer’s IP addresses.

First, participants were asked to give a short self-profile including their gender, semester, and previous knowledge of SAM and semantic tagging in general. Additionally, the participants were asked about their Internet usage habits for studying and private use.

The study comprised two parts. After a short introduction to SAM, the students were given four scenarios to explore their handling of SAM. Table 1 gives an overview on the scenarios covering the typical areas of application. The first two scenarios were given by two pictures to which the participants had to find keywords. This is a typical usage scenario for emedia skills lab staff when uploading new MLOs. The first scenario derived from the Nervous System Class, which is in the 4th semester curriculum, and therefore the topic was known to the participants at the time of study. In contrast, the second scenario derived from the Skin Class, which belongs to the 6th semester curriculum, and therefore the students were not familiar with the topic. In the third and fourth scenarios, there was a change of perspective. The students were supposed to search for available MLOs associated with a given lecture topic via SAM.

**Table 1:** Structure of the four scenarios.

Scenario	Content	User role	Text size
1	MRI image of a glioblastoma	Staff	81
2	Picture of an erythema chronicum migrans	Staff	124
3	Lecture topic “polyneuropathies”	Student	73
4	Lecture topic “patient with cough”	Student	79

The participants had to name keywords they would have used to describe the pictures for Scenarios 1 and 2 or keywords they would have clicked on in order to find linked media for Scenarios 3 and 4. Afterwards, 19 usability questions regarding the use of SAM and three free-text items for suggestions concerning the future improvement of SAM could be answered. The usability questions were Likert-scaled items with an option for “no answer” as well.

### Data Elicitation

SAM generated log files based on the static Internet protocol (IP) addresses used in the computer lab adopted for the study. Anonymity was ensured as it was not possible to infer the individual participant from the IP addresses of the semi-public computer lab. The completeness of a survey result was defined by a minimum of four search queries including all four given keywords of the scenarios. All clicks and search queries performed during the introduction of SAM, before the starting keyword (“glioblastoma”) for Scenario 1 was entered, counted as not assigned to a scenario. This analysis focused on the main interest: the top 10 keywords mentioned by the participants. But there is also a long-tail interest, which we covered in our analysis by showing random keyword examples occurring only once.

### Data Analysis

Data analysis by descriptive statistics relied on summary statistics and data visualization (scatter plots, bar charts, box plots). Due to the exploratory character of the study, visual inspection of data plots had to play an important part, while these plots provide more information regarding the nature of the relationship compared to the investigation of summary statistics only (eg, correlation coefficients). A scatter plot was used to investigate the relationship between the mean number of clicks per query and the number of queries submitted. A tag cloud was adopted as a means for assessing the occurrence of the top 10 keywords in a semi-quantitative way.

Qualitative analysis was carried out as follows. The open-questions feedback was analyzed by bottom-up qualitative text coding [28]. Two people read the users’ statements separately and assigned individual codes to the statements while reading the text for the first time. Afterwards, they ordered the codes and indexed the statements for a second time using the complete code set now. Afterwards, the two persons compared their code sets and agreed on a common set sometimes unifying synonymous terms then used to produce a consensus assignment of codes to statements. This step produced a semantically adjusted rate of conformance between the raters (semantically equivalent codes assigned vs not assigned to the same statements). Interrater reliability was measured by Cohen’s kappa. A synoptic rearrangement of statements assigned to the same (previously consented) code then provided the basis of a qualitative interpretation and summary of the feedback.

### Ethical Issues

There were no medical data collected nor any risk or disadvantage implied. All data were acquired anonymously at any time of the study. The participation in the study was strictly voluntary. The course participants were informed they could simply skip the study-related parts of the e-learning module of the EBM course with no effect on their learning achievement or course marks. Study participants received comprehensive information on the objectives and methodology of the study. The consent of the RWTH Aachen Faculty of Medicine system block coordinators was obtained in advance of the study.

### Software Tools

The study was executed through LimeSurvey 1.85+ [29], an online survey app that contained the scenario descriptions and the instructions on the tasks as well as the usability questions at the end. The data were entered via the Web interface of LimeSurvey and later exported for analysis. Descriptive statistics and box plots were produced by the program R 3.1.0 [30]. Bar charts and the randomization of the bottom 10 keywords were produced by Microsoft Excel 2010. For flowcharts, we used Microsoft PowerPoint 2010. The tag cloud was produced with the worditout website [31].

## Results

### Profile of Participants

**Figure 3:**
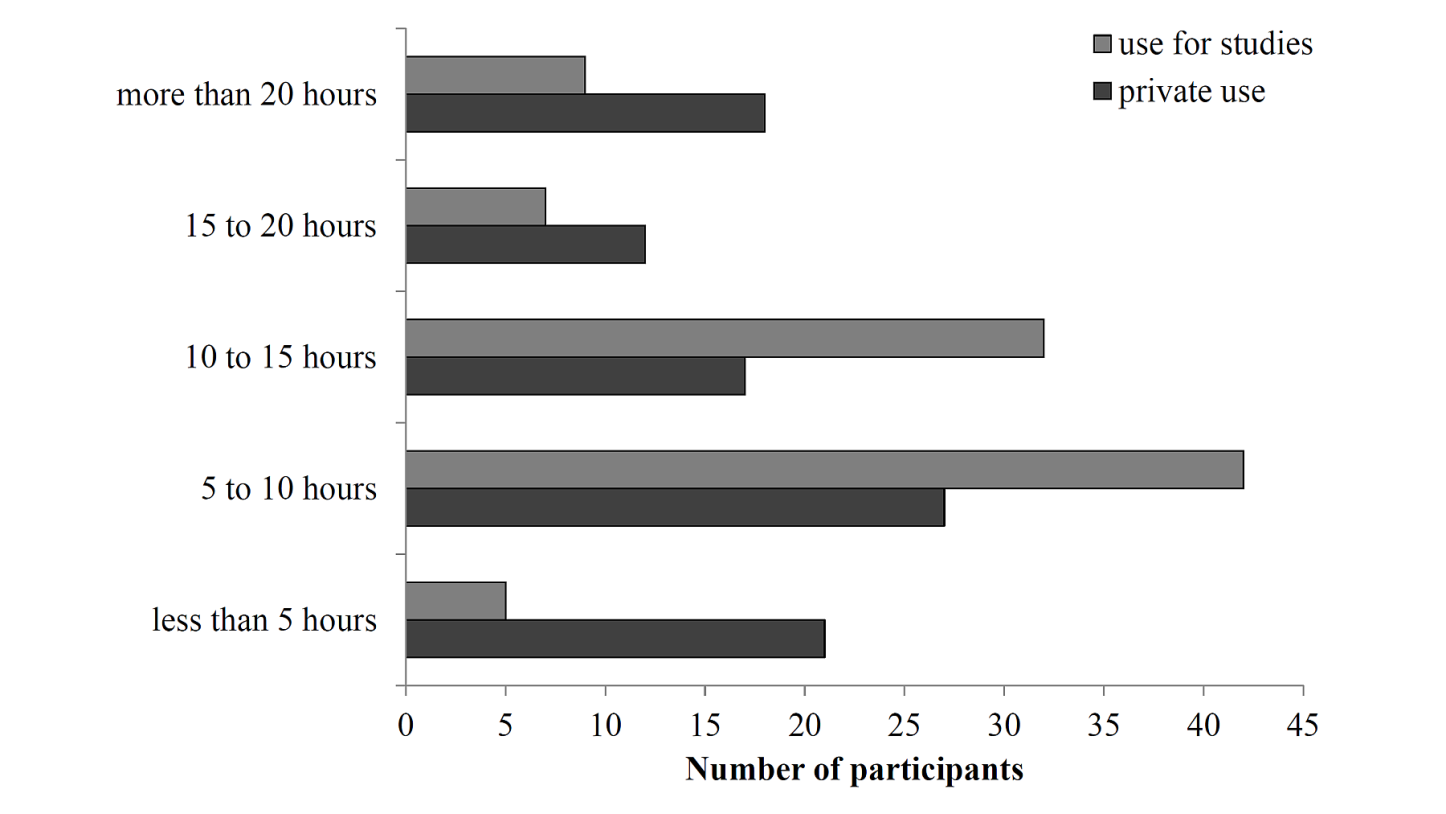
Internet usage habits of the participants (hours per week).

To investigate the semantic indexing of MLOs, we first analyzed the profile of participants and their Internet behavior. Table 2 and Figure 3 present the characteristics. We found a noticeable difference between Internet use for studies and private Internet use. The majority of the participants use the Internet 5-15 hours weekly for studying whereas for private use the hours vary between less than 5 hours and more than 20 hours weekly.

**Table 2:** Profile of all participants.

Characteristics		n
**Gender (N=95)**		
	Male	31
	Female	64
**Survey questions (N=95)**		
	I know about the emedia skills lab (yes)	87
	I have used the emedia skills lab before (yes)	75
	I know about tagging (yes)	17
	I have used tagging myself (yes)	8
**Completeness of the survey as given by LimeSurvey (N=101)**		
	Complete	95
	Incomplete	6
**Completeness of survey after analyzing the log files (N=101)**		
	Complete	90
	Incomplete	11

### Main Results

**Figure 4:**
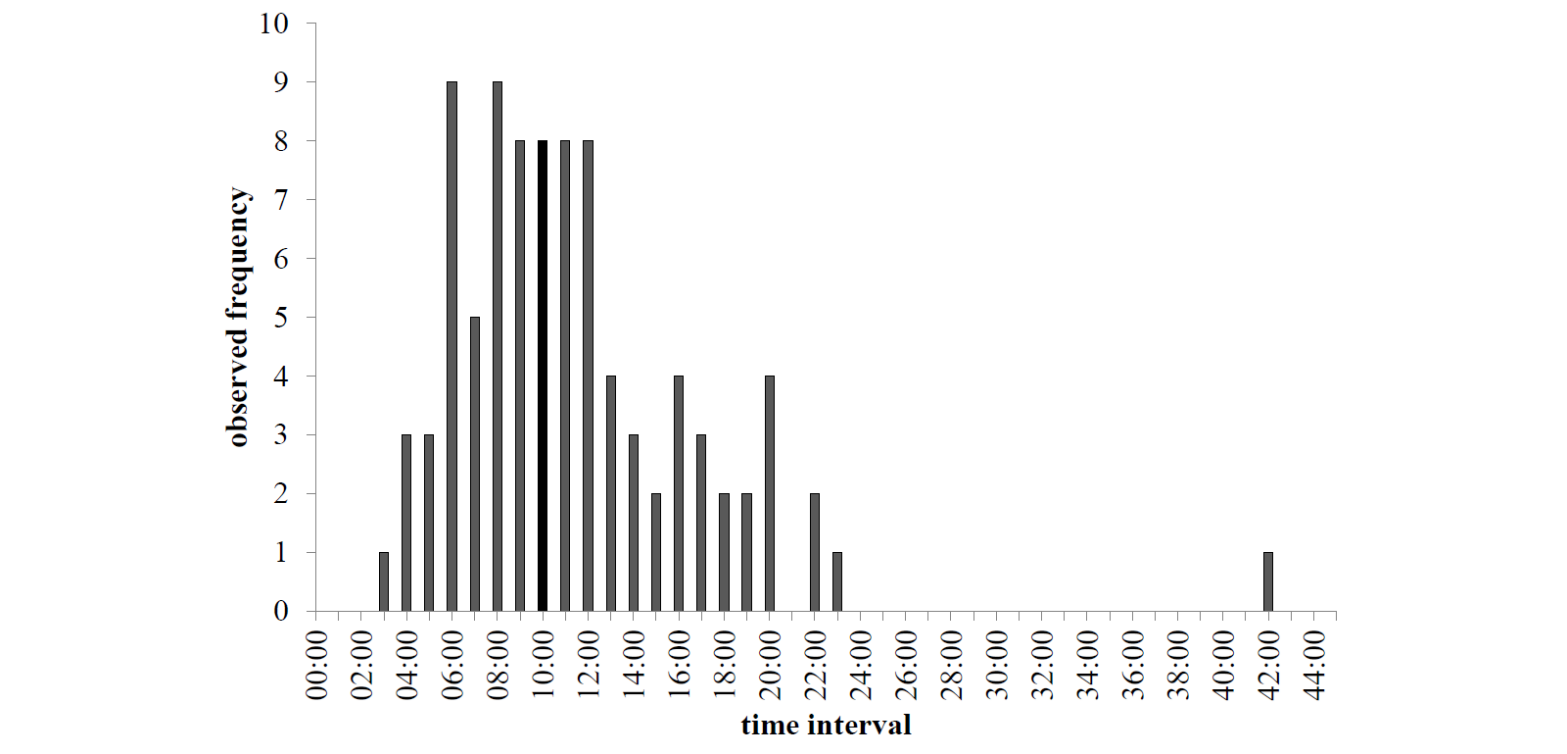
Observed frequency of duration to complete all four scenarios.

**Figure 5:**
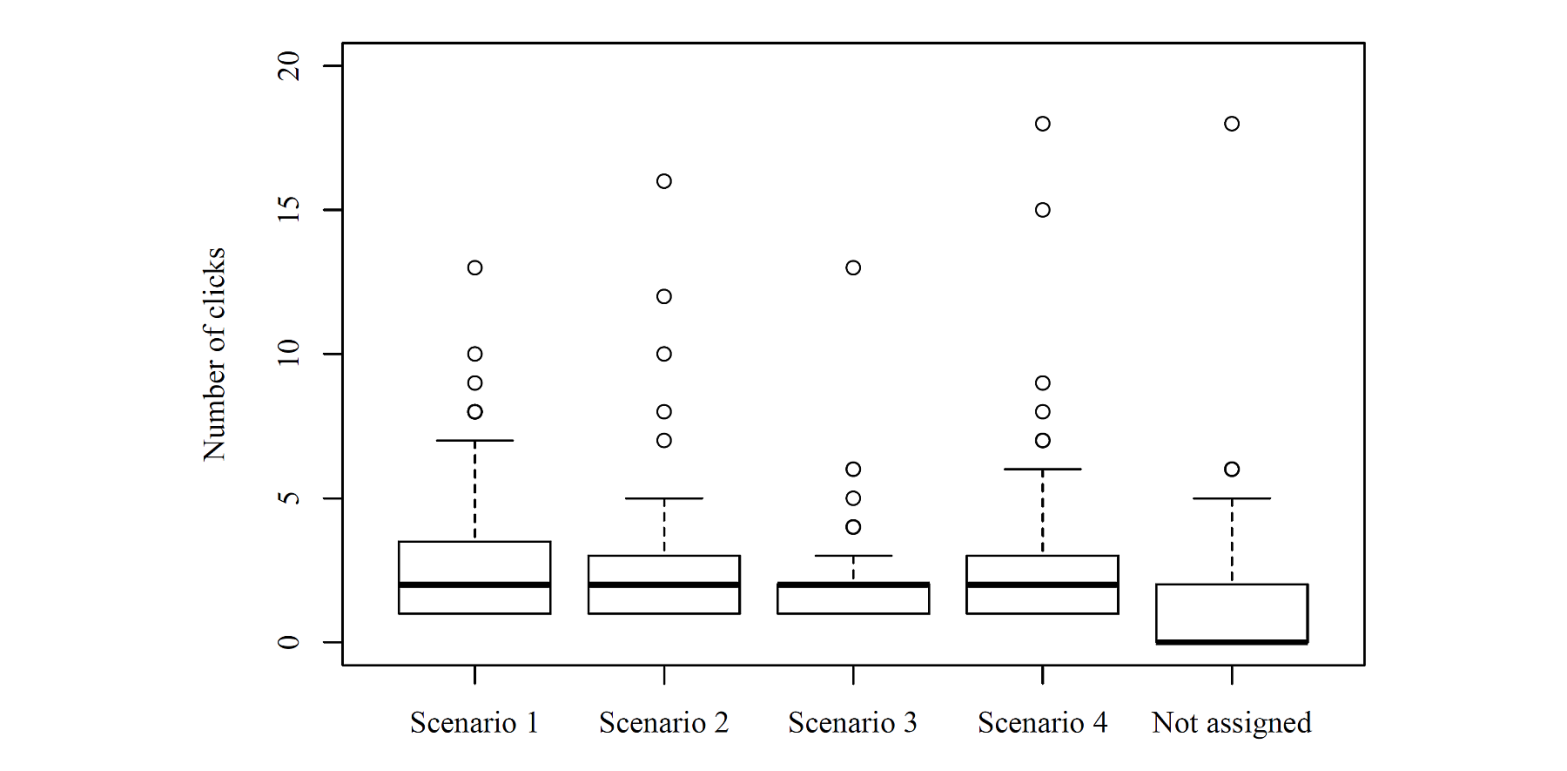
Number of clicks per scenario (x-axis: scenario; y-axis: number of clicks).

After the short self-profiling, the students were given four typical scenarios to explore their usage of SAM. We also monitored the time the students needed to complete all four scenarios in order to see whether or not they had enough patience to engage in the study and if they showed interest in SAM or not. The participants needed a total of 10.55 minutes on average (Figure 4). We analyzed the number of the students’ mouse clicks during the introduction and per scenario (Figure 5). Many students skipped the introduction (median 0) and started directly with the scenario, whereas few intensively familiarized themselves with SAM by using the introduction. For all four scenarios, the median number of clicks per scenario was two. Thus, there were no marked differences between the scenarios with respect to the total number of clicks, while for each scenario there was a wide spread in the number of clicks.

**Figure 6:**
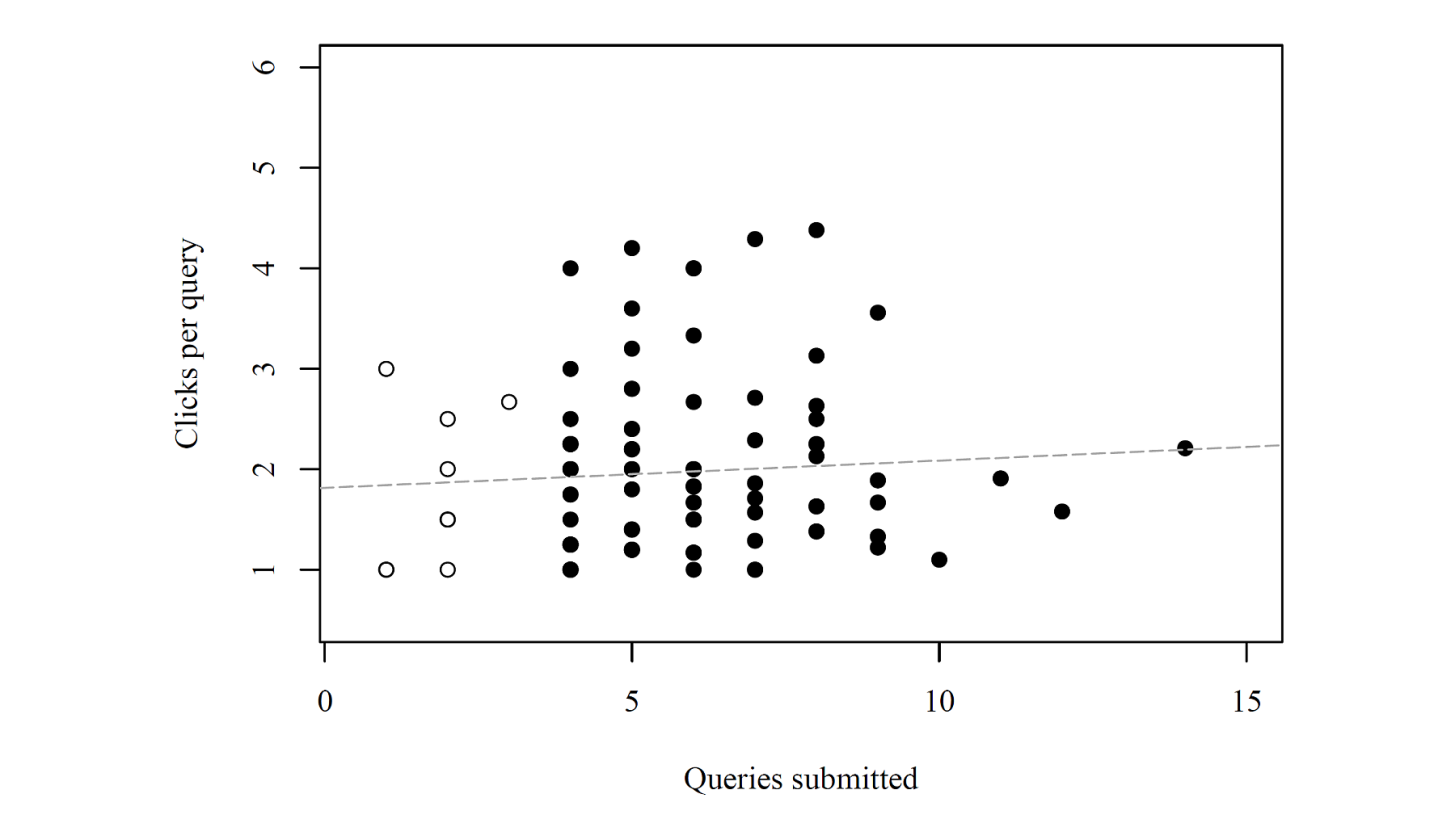
Correlation of clicks per query to queries submitted (empty circles: incomplete, filled circles: complete).

In addition to the total number of clicks per scenario, we investigated the relationship between the numbers of clicks per query and the number of queries. There could have been a linear or non-linear relationship (eg, well-oriented users could have used few queries, each accomplished by a few clicks, whereas disoriented users could have asked many unfocused queries, each accomplished by many exploratory clicks). Inspection of the scatter plot (Figure 6) of the mean number of clicks per query versus the number of queries yielded no marked relationship. The Pearson correlation coefficient was 0.06 indicating a lack of linear correlation. The empty circles represent the participants who did not complete all four scenarios, whereas the filled circles represent the participants that completed all of them.

### Keywords Given

Table 3 shows the total number of keywords entered for each scenario as well as the number of different keywords entered. For this report, we had to translate some of the German keywords into English. Since some participants entered the English synonym of the keywords up front, there is no distinction left between English and German keywords. Thus, we combined them in Table 3.

**Figure 7:**
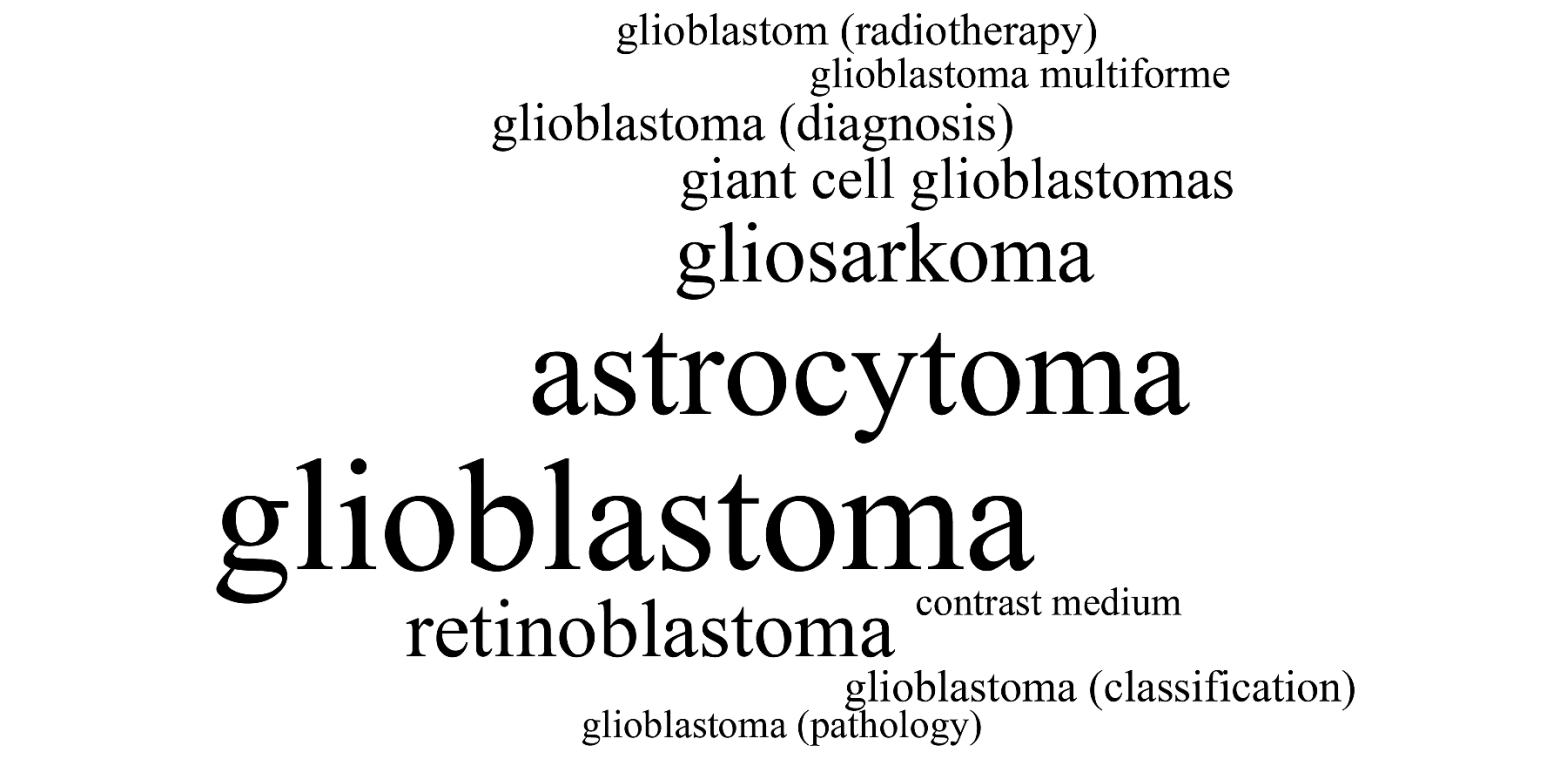
Tag cloud visualizing the top answers for Scenario 1.

Figure 7 shows an exemplary tag cloud visualizing the top 10 answers for Scenario 1. The larger the keyword is written, the more often it was mentioned by the participants for each scenario.

For Scenarios 1 and 2, 2 individual raters went through all keywords and rated them as either correct or incorrect according to the pictures given (Table 4). The data show a very high interrater reliability (Cohen’s kappa was .907 and 1 for Scenarios 1 and 2 respectively). Among the top 11 keywords mentioned for Scenario 1, there were 10 correct keywords and one incorrect. Among the top 11 keywords mentioned for Scenario 2, there were three correct and eight incorrect keywords.

Tables 5 and 6 give the 10 and 11 most frequently selected keywords, respectively, for the four scenarios. If two keywords occurred equally often, they share a rank. If the tenth place was tied, 11 keywords were listed. Also, 10 random examples of the long-tail keywords with only one nomination were listed.

**Table 3:** Keywords entered in each scenario.

	Total number of keywords entered	Number of different keywords entered
Scenario 1	346	74
Scenario 2	427	76
Scenario 3	394	102
Scenario 4	372	61

**Table 4:** Rating of Scenarios 1 and 2.

	Total	Correct	Incorrect	Inconsistent	Cohen’s kappa	*P* value
Scenario 1	74	49 (66.2%)	22 (29.7%)	3 (4.1%)	.907	5 × 10^-15^
Scenario 2	76	31 (40.8%)	45 (59.2%)	0 (0%)	1	0

**Table 5:** Top keywords used per scenario (Scenarios 1 and 2) and correctness of the keywords as assessed by 2 independent raters.

Rank		Keywords	Correctness	Occurrence
**Scenario 1**				
	1.	Glioblastoma	Correct	63
	2.	Astrocytoma	Correct	52
	3.	Gliosarkoma	Correct	28
	4.	Retinoblastoma	Incorrect	26
	5.	Giant cell glioblastomas	Correct	15
	6.	Glioblastoma (diagnosis)	Correct	12
	7.	Glioblastoma multiforme	Correct	11
	8.	Glioblastoma (classification)	Correct	9
	9.	Glioblastom (radiotherapy)	Correct	8
	10.	Glioblastoma (pathology)	Correct	6
		Contrast medium	Correct	6
	**Bottom ten (random examples) **			
		Magnetic resonance imaging	Correct	1
		Benign and malignant central nervous system neoplasms derived from glial cells	Incorrect	1
		Glioblastoma with sarcomatous component	Correct	1
		Glioblastoma (ethnology)	Incorrect	1
		Complications	Incorrect	1
		Glioblastoma (metabolism)	Incorrect	1
		Malignant form of astrocytoma	Correct	1
		Diagnosis	Incorrect	1
		Glioblastoma (ultrastructure)	Correct	1
		Grade IV astrocytomas	Correct	1
**Scenario 2**				
	1.	Erythema chronicum migrans	Correct	55
	2.	Larva migrans visceralis	Incorrect	33
	3.	Erythema infectiosum	Incorrect	25
	4.	Erythema	Correct	23
	5.	Larva migrans	Incorrect	22
	6.	Thrombophlebitis migrans	Incorrect	22
	7.	Lyme disease	Correct	22
	8.	Erythema induratum	Incorrect	19
	9.	Erythema nodosum	Incorrect	19
	10.	Erythema ab igne	Incorrect	17
		Benign migratory glossitis	Incorrect	17
	**Bottom ten (random examples) **			
		Skin disease, eczematous	Incorrect	1
		Erythema with elsewhere classified diseases	Incorrect	1
		Gyrate erythema	Incorrect	1
		Diagnosis	Incorrect	1
		Bullseye	Correct	1
		Erythema chronicum migrans (epidemiology)	Correct	1
		Bacterial lyme disease	Correct	1
		Chemically evoked	Incorrect	1
		Genetics	Incorrect	1
		Skin	Incorrect	1

**Figure 8:**
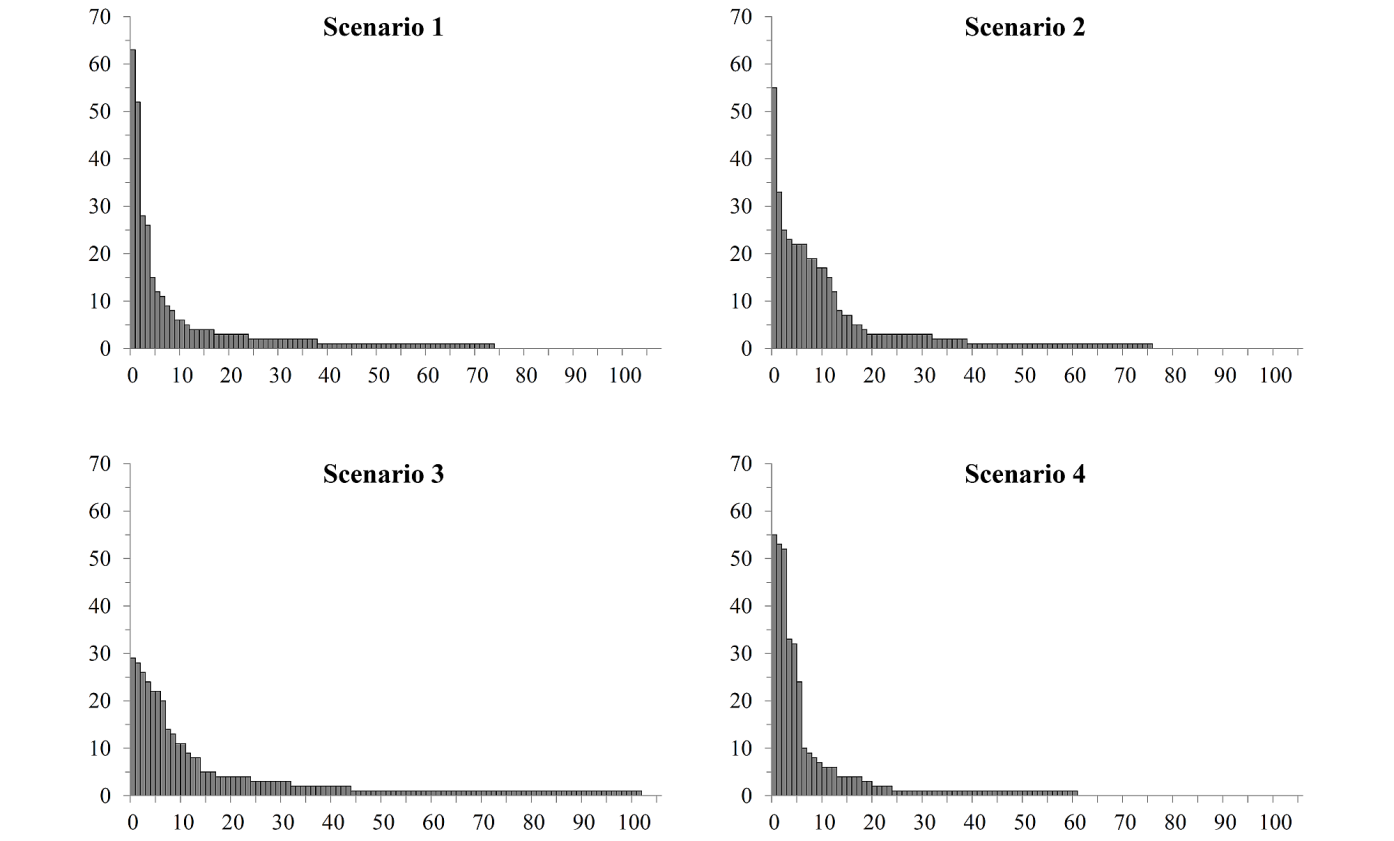
Distribution of keyword occurrence for the scenarios (x-axis: number of keywords given by students, y-axis: occurrence of one keyword).

Scenarios 1 and 4 show a power curve as expected in a long-tail figure, whereas Scenarios 2 and 3 still show a long tail at the end but a more bulky shape at the beginning (Figure 8).

**Table 6:** Top keywords used per scenario (Scenarios 3 and 4).

Rank		Keywords	Occurrence
**Scenario 3**			
	1.	polyneuropathies (PNP)	29
	2.	polyneuropathies (PNP), cause	28
	3.	polyneuropathies (PNP), diagnostics	26
	4.	subtypes of polyneuropathies (PNP)	24
	5.	other polyneuropathies	23
	6.	other specified polyneuropathies	22
	7.	polyneuropathies	20
	8.	peripheral nervous system, diseases	14
	9.	polyneuropathies (etiology)	13
	10.	peripheral nervous system	11
		polyneuropathies, (diagnosis)	11
	**Bottom ten (random examples)**		
		polyneuropathies critical illness	1
		peripheral nervous system, diseases, hereditary	1
		degeneration of the axon, myelin or both	1
		polyneuropathies (rehabilitation)	1
		diagnosis	1
		inherited polyneuropathy	1
		immunology	1
		symmetrical, bilateral distal motor and sensory impairment	1
		distribution of nerve injury	1
		disease of mulitneuronal nervs	1
**Scenario 4**			
	1.	symptoms, respiratory	55
	2.	breathing deficiency	53
	3.	cough	52
	4.	symptoms, that affect the circulatory and respiratory system	33
	5.	ambroxol	32
	6.	cardiac syncope	24
	7.	cough (etiology)	10
	8.	cough (therapy)	9
	9.	cough (diagnoses)	8
	10.	respiratory system	7
	**Bottom ten (random examples)**		
		hemoptysis	1
		other polyneuropathies	1
		influenza	1
		dyspnea	1
		mortality	1
		diseases of the respiratory system	1
		parasitology	1
		polyneuropathies (PNP), causes	1
		cough (mikrobiology)	1
		glottis	1

### Results of Usability Questions

**Figure 9:**
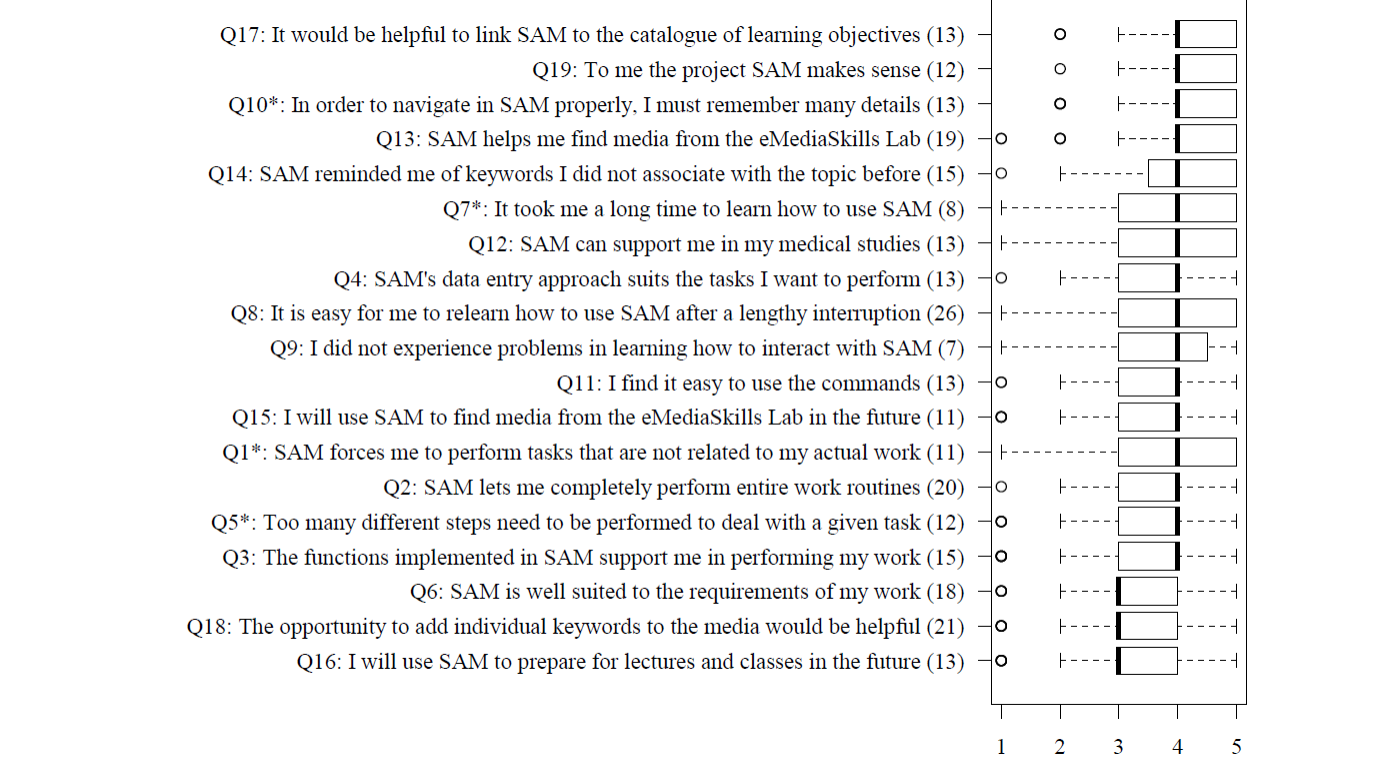
Usability questions in order of approval on a Likert Scale of 1-5 (5 best, 1 worst). The number of “no answers” is denoted in parentheses. Items yielding negative implications for the rating of the systems (marked by *) are given in the original wording, while the scale was inverted in the figure (thus, a high rating represents a positive appraisal).

Figure 9 shows the results of the usability questionnaire. Overall, the participants rated the usability of SAM positively (median 4 for nearly all questions). They clearly marked the advantages of finding new keywords and the search feature for the emedia skills lab. The students stated that the easy handling and fast learnability of SAM were positive features. However, at this stage of SAM, with only a few media linked to it, most participants were indifferent about their future use of SAM for preparing for lectures and classes. They were also indifferent about the possibility of adding individual keywords to SAM (median 3). A few questions seemed to be hard to answer for the students as more than 20% of the participants chose “no answer” as an option. These questions dealt with repeated uses of SAM and the possibility of adding individual keywords to the media. At the time of the study, the students did not have any routine for using SAM.

### Free-Text Items

Two raters who evaluated the free-text items assigned 81 codes to the statements given. The sets of the 2 raters conformed on a large scale: Cohen’s kappa was .857 (z=34, *P*=0) for their independent rating. For the variations, they found a consent code together (eg, “user-friendliness” vs “usability” consented as “usability”).

Most positive feedback was given about the clarity of SAM (Table 7). Usability and simplicity also had strong support. These keywords refer to the general user interface of SAM. Other frequently addressed attributes are synonyms and new associations, which refer more to the semantic content of SAM.

**Table 7:** “What did you like best?” positive feedback free-text items (codes: given by raters, frequency: of appearance, representative statement: selected typical statement).

Codes	Support	Representative statement
Clarity	9	The clarity
Usability	7	User-friendly interface
Simplicity	7	Easy principle, self-explanatory
Synonyms	6	The big group of linked keywords
Associations, new	5	Keywords come up that I have not associated with the search term before
Search feature	3	Fast search for distributed media
Media	2	The pictures
Knowledge structure	1	Organization and structure of knowledge
Information access, easy	1	Good idea to link information to have an easier access. Thanks

Negative feedback was given about unclear associations where participants were not able to understand associations given by SAM (Table 8). This might also be related to the complaints about missing definitions of keywords and complicated navigation. Another negative aspect mentioned often was the missing media in many categories. Two participants pointed out that the search feature is not very advanced since one can search only for keywords included in SAM. SAM is mainly in German, especially the MeSH keywords. Only the definitions and synonyms are in English. However, 2 participants were irritated by the English language.

**Table 8:** “What did you disapprove of?” negative feedback free-text item (codes: given by raters, frequency: of appearance, representative statement: selected typical statement).

Codes	Support	Representative statement
Associations, unclear	5	In some keywords I did not see the association with the search term
Media, missing	5	Nonexistent media so many categories were useless
Previous knowledge	2	The keywords are only usable with advanced knowledge
No German	2	SAM is in English although it is for German students. Why is the standard language not German combined with an international offer?
Navigation, complicated	2	Navigation is a bit complicated
Definitions, missing	2	The definition of the keywords is insufficient
Search feature	2	No possibility of searching for individual keywords
Introduction not sufficient	1	The introduction could not sufficiently show SAM’s potential. It is rather confusing
Presentation of results	1	The way of presenting the search results
Time-consuming	1	The constant redirecting is time-consuming
SAM unknown to user	1	I did not know SAM until today
No advantage	1	It is not distinct from other search engines, so I am not sure if I will use SAM in the future
Associations, missing	1	Missing link to differential diagnoses and diagnostic options of diseases

The top suggestion is to add further media to SAM that relate to the negative feedback about missing media (Table 9). Other participants suggested general further development of SAM that might include the missing media or search functionality mentioned above in the negative feedback.

**Table 9:** “What suggestions do you have?” suggestion feedback free-text item (codes: given by raters, frequency: of appearance, representative statement: selected typical statement).

Codes	Support	Representative statement
Media, enhancement	4	Links to more media
Further development	3	Further development of the project
Definition of keywords	1	Short definitions behind keywords
Link to curriculum	1	Link to curriculum and information about relevance ranking according to your level of education
German language	1	Articles in German
Promote SAM	1	More promotion of SAM, I have not heard of it before
Connect e-learning platforms	1	Connection to other e-learning platforms with help of keywords, ie L2P
Better introduction	1	Better exercises to get to know SAM
Navigation	1	Navigation bar

## Discussion

The study aimed to explore typical usage patterns of students who use SAM for browsing, searching, and indexing MLOs and to assess the usability of SAM focusing on (1) the user interface and (2) the suitability of the currently used SN for accessing and indexing MLOs. As stated in the introduction, there is a lack of usability assessment in medical e-learning [23].

### Profile of Participants

A total of 95 participants completed the survey, while (according to the log files) only 90 participants completed all four scenarios. We surmise that participants might have copied the keywords from their neighbors or just made up some keywords, and we cannot completely exclude that some participants might have finished the survey later when their activities were no longer recorded.

### Main Results

The average time for completing the study and the respective spread showed satisfactory commitment (enough time on average for adequately addressing the task) and a broad variation of user behavior. However, there was 1 participant who took 42 minutes to complete the study, which could be explained by extraordinary thoroughness or a long time needed to familiarize with SAM. Alternatively, he might have been distracted in between and returned later to finish the survey. As shown by the respective numbers of clicks not assigned to scenarios, a relatively small number of students took time to familiarize themselves with SAM. Based on the similar median, the scenarios can be assumed to be equally interesting and equally hard to work on. Moreover, the analysis of duration and click rates shows that, on average, the students worked quickly without browsing through a lot of other keywords. However, there is a wide spread, so there were also a few students that followed a browsing approach instead of a more focused keyword access. The analysis shows that there is no marked relationship between the mean number of clicks per query and the number of queries, which simply illustrates the broad variability of search behavior not being obviously determined by single factors.

### Keywords Given

In Scenario 1, some students might not have been familiar with the topic, although it had already been part of their curriculum. Also, some participants might have entered random keywords because they wanted to finish the tasks quickly. This could explain the 22 incorrect keywords. According to the curriculum, the students had not come across the Scenario 2 topic at the time of the study, which explains the number of wrong keywords. Most of the top 11 keywords mentioned for Scenario 2 show up in the associated keywords list of SAM after the search in SAM for “erythema chronicum migrans”. Nonetheless, the scenario is realistic in some sense. Usually student assistants working for the emedia skills lab are responsible for uploading and indexing the media. Our results underline the need for a profound knowledge of SAM’s SN as well as the content of the media when adding new MLOs to the emedia skills lab and indexing it. The quality of indexing the uploaded media could be improved by hiring students from higher semesters and giving them a detailed introduction and training of SAM before they start their jobs as student assistants for the emedia skills lab. Also, it is not clear if medical teaching staff were better at indexing MLOS since their familiarity with the content of the media might not compensate for their being unfamiliar with SAM’s SN.

For Scenarios 3 and 4, the keywords were not classified as correct or incorrect since the students were free to enter keywords of their choice to find additional media. Therefore, the long tail for Scenarios 3 and 4 could indicate a broad interest in different topics as well as different previous knowledge of the individual participants. Scenario 3, in particular, shows a broad interest (102 keywords for one single scenario). Nevertheless, some students might have entered random keywords to finish the assignment quickly. Given the structure and purpose of SAM, no gold standard outcome for Scenarios 3 and 4 could be specified. A scenario in which students would have to find a certain MLO would not represent a realistic use of SAM. Nonetheless, the definition of usability does not necessarily imply the existence of a unique solution to a given problem—otherwise no device for exploring or managing an information space given an individual perspective or interest could be assessed with respect to its usability. Nonetheless, maintaining a focused search and avoiding disorientation while using a complex information space can be considered “achieve[ing] specified goals with effectiveness, efficiency, and satisfaction” (see the earlier definition of usability [14]), and our results at least indicate that the majority of students succeeded here. Additionally, the usability questions adapted from IsoMetrics were adjusted to these circumstances.

In all four scenarios, we observed that besides the top 10 keywords, there are many related keywords that rarely occur directly in connection with the starting keyword. We interpret the less frequently named keywords as long-tail interest. SAM supports linking top keywords to long-tail keywords, which the students may not have associated with a given topic while using regular learning strategies.

### Usability Questions

This study was based on the IsoMetrics usability inventory. Hamborg et al showed that IsoMetrics is a reliable technique for software evaluation in the field of hospital information systems [32]. While hospital information systems often use tools for indexing medical diagnoses with keywords, this result motivates the use of IsoMetrics in the context of our study. Also the results of Hamborg yielded a better outcome when he compared the IsoMetrics usability inventory to usability testing methods [33]. Nevertheless, many questions of the IsoMetrics questionnaire could not be adapted to SAM or were not in the focus of this study, which is not a mere software evaluation but rather a study to investigate the acceptance, use, and usability of an SN in medical e-learning.

### Free-Text Items

Some of the improvement suggestions go along with the negative feedback. Missing media were often mentioned. New media are continuously added to the emedia skills lab, so the situation can be expected to improve in the future.

One suggestion was to promote SAM in lectures. Obviously, the more faculties know about SAM and provide new media, the more the teachers will talk about SAM in their lectures and classes. Another suggestion was to link SAM’s keywords with the curriculum and level of education. A connection between SAM and the catalogue of learning objectives is already planned for the future. One remark was about the lack of German definitions of the MeSH terms. At present, only the MeSH terms are officially translated into German by the Deutsches Institiut für medizinische Dokumentation und Information (DIMDI), but not the annotations, cross references, and definitions included in the English MeSH version [34]. Therefore, the definitions were taken from the English version of MeSH.

### Comparison With Prior Work

Willett et al identified the need for a standardized taxonomy for medical education and created a taxonomy called TIME-Indexing (Topics for Indexing Medical Education) [35] that can be used for indexing MLOs as well as for curricular mapping. So far, the usability of TIME-Indexing has not been investigated. However, Willet highlights the need for research on ontology use in medical education [36].

Blaum et al performed a systematic review of 14 different structured vocabularies for characterization of MLOs to be organized in an SN [37]. According to the authors, none of those proved to be a good content description of medical curricula and learning resources in the German-speaking world. Neither work systematically investigated the usage of the taxonomy or vocabulary, nor did the authors address usability aspects of the respective computer applications.

SAM visualizes the concept space and helps in navigating it. Cimino introduced a network of concepts called “concept space” [11]. The approach was used, for example, by Karlsson et al to develop a medical decision support system using a controlled medical terminology [38]. Later on, Berners-Lee developed the idea of the Semantic Web [39]. The Semantic Web focuses on automatic interpretation of the World Wide Web and not on the reduction of cognitive overload. SAM focuses on an easier navigation and also the reduction of cognitive overload.

In other areas of application, ontologies and semantic Webs are visualized in different ways. For example, Le Grand and Soto discussed different visualization ideas of topic maps, including graphical three-dimensional representations based on virtual reality concepts like computer-generated landscapes and virtual cities [40]. In comparison to that, SAM so far presents only a list of concepts. In the future, a more sophisticated graphical representation of SAM might be helpful for the users, especially since some participants disapproved of the complicated navigation and unclear associations in the free-text items. Katifori et al gave an overview on different ontology visualization methods and tools [41]. These tools are not only intended to navigate ontologies but also to model and administer them.

New MLOs of the emedia skills lab are indexed manually with SAM. There is also some research on semi-automatic indexing. Gay et al developed an extension of an existing tool, the Medical Text Indexer, to semi-automatically tag biomedical articles [10]. Lacoste et al developed an intermedia medical image indexing and retrieval system [42]. Semi-automatic indexing will be hard if not impossible for videos and other interactive MLOs that are uploaded to the emedia skills lab.

### Limitations

While the study focuses on usability aspects, it does not establish a comparative design addressing the way the participants work with, versus without, SAM and their respective effort or efficiency to retrieve learning media. The effects of SAM on the learning outcome of the students were not investigated, while the design of the study does not allow the overall benefit of SAM to be measured. Few incomplete answers had to be excluded from the results in our study.

The interpretation of the long-tail results was complicated by similar keywords being entered leading to a larger number of keywords. The top 11 answers in Scenario 1, for instance, include many keywords containing the word “glioblastoma”. Some were enhanced by MeSH qualifiers, while others are more specific, for example, “glioblastoma” versus “glioblastoma multiforme”. A similar pattern can be seen in Scenario 2. One keyword among the top 11 is “erythema chronicum migrans”; the other one is just “erythema”. Therefore, not all of the different keywords entered in each scenario are semantically independent.

### Conclusions

Our study demonstrated that it is possible to develop a Web-based tool for indexing and retrieval of MLOs that yields to high rates of user satisfaction and user commitment representing important aspects of usability. For the first time, we analyzed the typical usage pattern and habits of using an SN for indexing and accessing MLOs. Introducing a controlled vocabulary or classification system generally homogenizes the indexing but requires preliminary training of the indexers. SAM succeeded in a similar homogenization but also yielded a spectrum of individual associations in the long tail as it is typically found when applying Web 2.0 structures. A long-tail interest in random topics can be supported by SAM. We found SAM to be a tool with high acceptance and usability that can be used as a tool for many MLO repositories in different locations.

## Abbreviations

DIMDI: Deutsches Institiut für medizinische Dokumentation und Information (German Institute for Medical Documentation and Information)
EBM: evidence-based medicine
ICD: International Classification of Diseases
ISO: International Organization for Standardization
IP: Internet protocol
MeSH: Medical Subject Headings
MLOs: medical learning objects
SAM: Semantically Annotated Media
SKOS: Simple Knowledge Organization System
SN: semantic network
TIME-Indexing: Topics for Indexing Medical Education
UMLS: Unified Medical Language System
